# Reconstruction of an anterior chest wall defect in a child using a latissimus dorsi muscle-thoraco-lumbar fascia composite flap – A case report

**DOI:** 10.1016/j.ijscr.2020.06.004

**Published:** 2020-06-12

**Authors:** Mohammad M. Al-Qattan, Waseem M. Hajjar

**Affiliations:** a Division of Plastic Surgery, Department of Surgery, King Saud University, Riyadh, Saudi Arabia; b Division of Thoracic Surgery, Department of Surgery, King Saud University, Riyadh, Saudi Arabia

**Keywords:** Reconstruction, Chest wall, Children, Latissimus dorsi muscle-thoraco-lumbar fascia composite flap

## Abstract

**Introduction:**

Reconstruction of chest wall defects in children poses a challenge because the use of hard implants will impair chest wall growth. In this report, we demonstrate the reconstruction of a chest wall defect in a pediatric patient with an innovative technique using the latissimus dorsi muscle-thoraco-lumbar fascia composite flap.

**Case Report:**

A 5-year old boy presented to the clinic with a congenital anterior right chest wall defect. Reconstruction was done using this composite flap. Drill holes were made in the sternum and remaining ribs at the edges of the chest wall defect. The muscle-fascia composite flap was then sutured over the defect using polypropylene sutures anchored to these drill holes. There were no post-operative complications. At final follow-up 11 years later, the chest wall depression was still present but to a lesser degree when compared to the pre-operative depression. There was no bulging of the lung through the defect.

**Discussion:**

The composite flap technique was compared to other options of chest wall reconstruction in children.

**Conclusions:**

Congenital anterior chest wall defects that are not associated with breathing problems may be reconstructed in early childhood with a pedicled latissimus dorsi muscle-thoraco-lumbar fascia composite flap. The flap is rigid enough to prevent bulging of the lung though the defect and hence, it provides an adequate reconstruction. However, the remaining chest wall depression and further chest wall rigidity requires a second operation after puberty.

## Highlights

• Reconstruction of chest wall defects in children poses a challenge because the use of hard implants will impair chest wall growth.
• We demonstrate the reconstruction of a chest wall defect in a pediatric patient with an innovative technique.
• At final follow-up 11 years later, there was no bulging of the lung through the defect.
• The case demonstrates that the composite flap is rigid enough to prevent bulging of the lung though the defect.

## 1 Introduction

Reconstruction of chest wall defects in adolescents and adults is usually done using implants. Pre-designed implants using fine-cut computed tomography may also be obtained for a ‘perfect’ fit into the defect [1]. In contrast, reconstruction of chest wall defects in children poses a challenge because the use of hard implants will impair chest wall growth. The standard technique of reconstruction in these pediatric cases has been by the use of an artificial absorbable material (such as biodegradable collagen scaffolds) which is supported with an autogenous adjacent rib ‘swinged’ over the absorbable material for support [2].

In this report, we demonstrate the reconstruction of a chest wall defect in a pediatric patient with an innovative technique using the latissimus dorsi muscle-thoraco-lumbar composite flap. The lower part of the thoraco-lumbar fascia is very thick and strong. It is adherent to the under surface of the lower part of the latissimus dorsi muscle, and fuses with the aponeurotic origin of the muscle from the iliac crest. We show in our case that the incorporation of this fascia with the muscle flap provides adequate reconstruction (without the need for an overlying rib support) and prevents the bulging of the lung through the defect. The work has been reported in line with the SCARE criteria [3].

## 2 Case report

**Fig. 1:**
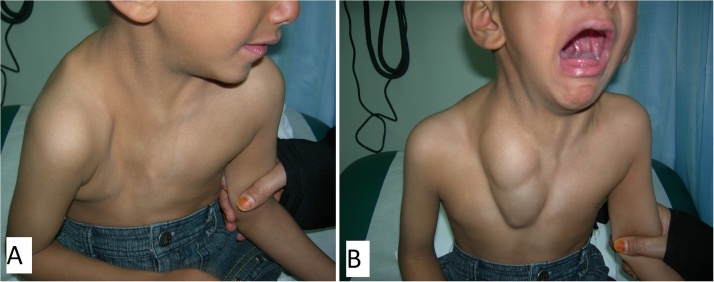
Preoperative views at age of 5 years. A) Upon inspiration, B) The tense and bulging lung under the skin while crying.

**Fig. 2:**
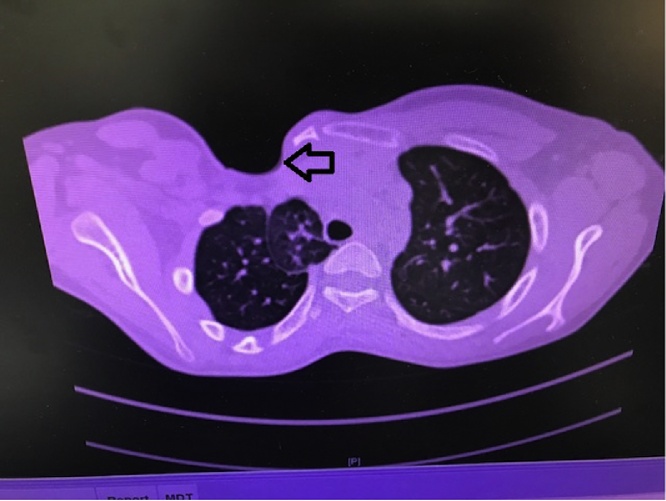
Preoperative CT Scan showing the skin over the pleura with no muscles across the defect (the arrow points to the defect).

**Fig. 3:**
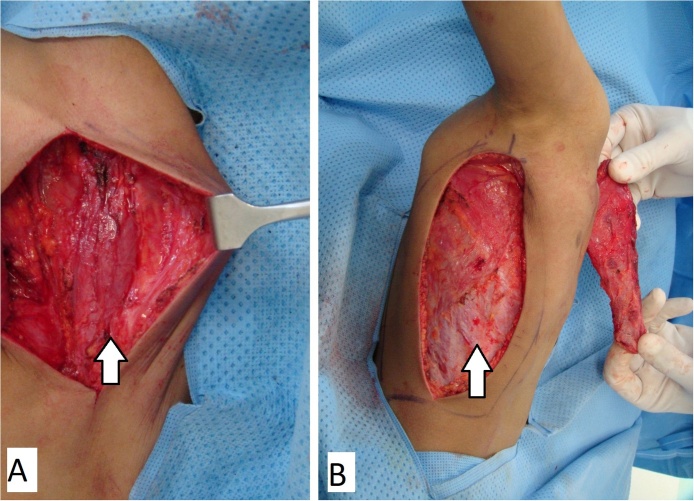
Intraoperative views. A) Dissection of the skin from the pleura anteriorly. The arrow points to the exposed pleura. B) The latissimus dorsi muscle-thoraco-lumbar fascia composite flap has been dissected and tunneled anteriorly to the defect (the arrow points to the area where the thoraco-lumbar fascia has been removed).

**Fig. 4:**
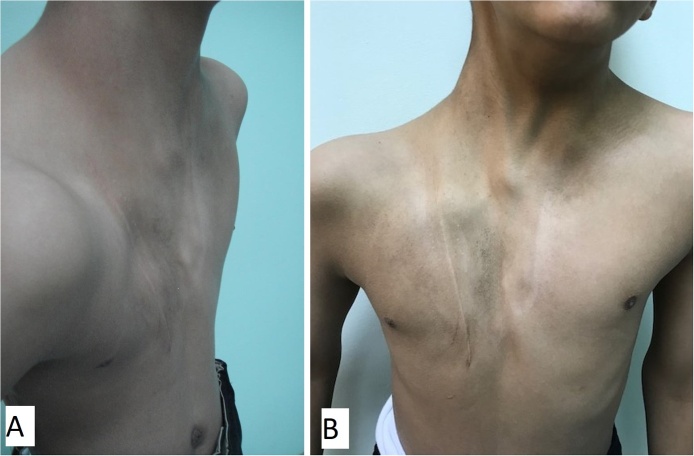
Postoperative views at age of 16 years. A) upon inspiration, B) upon expiration. Note that there is no bulging of the lung.

**Fig. 5:**
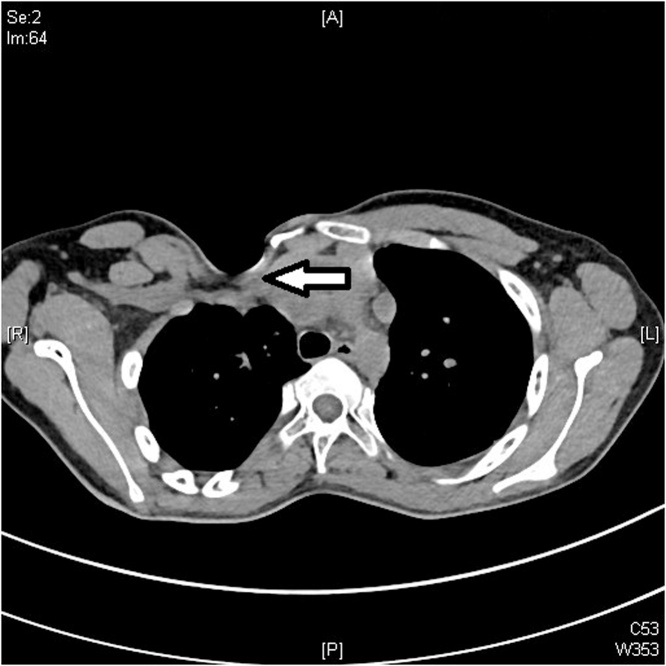
Postoperative CT scan showing the thick composite flap over the pleura (arrow).

A 5-year old boy presented to the clinic with a congenital anterior right chest wall defect. The delivery was by normal vaginal delivery after a full-term pregnancy. There was no breathing difficulty at birth, and no history of paradoxical breathing. There were no other congenital abnormalities, and development was normal. Medical advice was taken from his local town and this child was referred to our center at age of 5 years. Examination showed an otherwise healthy child with an anterior right chest wall defect. There was no paradoxical breathing and oxygen saturation at rest was 99%. There was no history of breathing problems even with exercise. The clavicle was absent but the sternum was intact. The anterior rib defect extended from the first to the fifth rib. The defect was felt upon inspiration (Fig. 1A) and the right lung protruded through the bony defect upon expiration; and the bulge became more prominent and tense when crying (Fig. 1B). A CT scan showed the defect with the skin adherent to the pleura. There were no pectoral muscles across the defect (both the pectoralis major and minor were absent) (Fig. 2). Surgery was done by a team of plastic and thoracic surgeons. The patient was put in the semi-lateral position. A longitudinal skin incision was made anteriorly over the chest wall defect and care is given not to injure the pleura while dissecting off the skin (Fig. 3A). Another longitudinal skin incision was made over the latissimus dorsi muscle. The muscle was dissected off based on the neurovascular bundle. The thoraco-lumbar fascia was incorporated with the lower part of the muscle. The composite flap was then tunneled to the anterior chest wall defect as a pedicled flap (Fig. 3B). Drill holes were made in the sternum and remaining ribs at the edges of the chest wall defect. The muscle-fascia composite flap was then sutured over the defect using polypropylene sutures anchored to these drill holes. Two drains were inserted (one anterior drain and one posterior drain), and the skin was closed with absorbable sutures. There were no post-operative complications. At final follow-up 11 years later, the chest wall depression was still present but to a lesser degree when compared to the pre-operative depression. There was no bulging of the lung through the defect upon expiration, exercise or crying (Fig. 4A, B). A CT scan showed the thick muscle-fascia composite flap over the pleura (Fig. 5). The patient was offered implant coverage at age of 18 years, but the parents refused and stated that they will think about it.

## 3 Discussion

Our algorithm of management of congenital chest wall defects depends on the presence of paradoxical breathing/breathing difficulties. If present, early surgery in the neonatal period is indicated. The use of latissimus dorsi muscle in this age is difficult and the muscle is not yet well-developed. Therefore, reconstruction of the defect in the neonatal period is done using a collagen matrix and the chest is supported by the swinging rib technique [2]. If there is no paradoxical breathing or breathing difficulties, we delay the reconstruction till the age of 5 years. We utilize the latissimus dorsi muscle-thoraco-lumbar fascia composite flap technique. This composite flap technique has not been previously reported in the literature. We show in our case that the incorporation of this fascia with the muscle flap provides adequate reconstruction (without the need for overlying rib support) and prevents the bulging of the lung through the defect. After puberty, the use of an implant will correct the residual depression and will provide the rigid support [1].

There are several technical points that need to be taken in consideration to ensure a successful reconstruction. Careful dissection of the skin form the pleura is mandatory, and a chest tube needs to be stand-by during the procedure. Hence, surgery should be performed by a team of plastic and thoracic surgeons. The nerve to the latissimus dorsi muscle should be preserved and included in the neurovascular pedicle in order to minimize the post-operative muscle atrophy. Suturing of the muscle-fascia composite flap to the edges or the defect should be done with the muscle stretched, and sutures should be done through the surrounding bone.

The main question is regarding alternatives to our composite flap technique. One might argue that collagen matrix reconstruction is less invasive. We believe that the use of collagen matrix alone does not provide a rigid enough layer over the lung to prevent the bulging of the lung through the defect. Hence, the use of swinging ribs is needed [2]. Other advantages of our composite flap reconstruction over the collagen matrix reconstruction include cost-effectiveness and the theoretical lower risk of post-operative infection because of the use of autologous tissue. In addition to the collagen matrix, other materials are available and has been used. An example is the use of a non-absorbable mesh [4,5]. However, some restriction of the growth of the chest wall will be expected. Other materials include dural patches [6], absorbable patches and xenografts [7]. Once again, these absorbable materials are not rigid enough and require bone support using the swinging rib technique.

## 4 Conclusions

Congenital anterior chest wall defects that are not associated with breathing problems may be reconstructed with a pedicled latissimus dorsi muscle-thoraco-lumbar fascia composite flap. The flap is rigid enough to prevent bulging of the lung though the defect and hence, it provides an adequate reconstruction. However, the remaining chest wall depression and providing chest wall rigidity requires a second operation after puberty.

## Funding

None.

## Ethical approval

The study was approved by the research committee, National Hospital (Care), Riyadh, Saudi Arabia.

## Consent

Written informed consent was obtained from the father of the patient for publication of this case report and accompanying images. A copy of the written consent is available for review by Editor-In-Chief of this journal on request.

## Authors’ contribution

Both authors contributed significantly and in agreement with the content of the manuscript. Both authors participated in data collection and in writing of the manuscript. The first author headed the plastic surgery team and the second author headed the thoracic surgery team.

## Registration of research studies

Not relevant here.

## Guarantor

M.M. Al-Qattan.

## Provenance and peer review

Not commissioned, externally peer-reviewed.

## Declaration of Competing Interest

None.
